# Identification of Prostaglandin Pathway in Dinoflagellates by Transcriptome Data Mining

**DOI:** 10.3390/md18020109

**Published:** 2020-02-13

**Authors:** Valeria Di Dato, Adrianna Ianora, Giovanna Romano

**Affiliations:** Department of Marine Biotechnology, Stazione Zoologica Anton Dohrn Napoli, 80121 Napoli, Italy; adrianna.ianora@szn.it (A.I.); giovanna.romano@szn.it (G.R.)

**Keywords:** dinoflagellates, prostaglandins, transcriptomes, bioinformatics, FPKM, PUFA, marine microalgae, stress, bioactive compound, MMETSP.

## Abstract

Dinoflagellates, a major class of marine eukaryote microalgae composing the phytoplankton, are widely recognised as producers of a large variety of toxic molecules, particularly neurotoxins, which can also act as potent bioactive pharmacological mediators. In addition, similarly to other microalgae, they are also good producers of polyunsaturated fatty acids (PUFAs), important precursors of key molecules involved in cell physiology. Among PUFA derivatives are the prostaglandins (Pgs), important physiological mediators in several physiological and pathological processes in humans, also used as “biological” drugs. Their synthesis is very expensive because of the elevated number of reaction steps required, thus the search for new Pgs production methods is of great relevance. One possibility is their extraction from microorganisms (e.g., diatoms), which have been proved to produce the same Pgs as humans. In the present study, we took advantage of the available transcriptomes for dinoflagellates in the iMicrobe database to search for the Pgs biosynthetic pathway using a bioinformatic approach. Here we show that dinoflagellates express nine Pg-metabolism related enzymes involved in both Pgs synthesis and reduction. Not all of the enzymes were expressed simultaneously in all the species analysed and their expression was influenced by culturing conditions, especially salinity of the growth medium. These results confirm the existence of a biosynthetic pathway for these important molecules in unicellular microalgae other than diatoms, suggesting a broad diffusion and conservation of the Pgs pathway, which further strengthen their importance in living organisms.

## 1. Introduction

Fatty acids in general and PUFAs in particular have essential functions in living organisms from all taxa, ensuring fundamental homeostatic functions [1]. Their presence is needed for the maintenance of membrane fluidity and other functions such as communication and defence, correct functioning of the cardiovascular and nervous systems, immunity and inflammation-related responses [1,2,3,4,5,6,7]. 

Marine eukaryotic microorganisms represent a good source of PUFAs, including long chain ω3- and ω6-PUFAs such as eicosapentaenoic acid (EPA), eicosatrienoic acid (ETrA), docosahexaenoic acid (DHA) and arachidonic acid (ARA). These are of particular interest for human health since they are beneficial for the prevention of cardiovascular diseases and chronic inflammation [1]. Microalgae, in particular diatoms, cryptophytes and dinophytes, are good producers of EPA, ETrA and DHA, but less of ARA [1,2,3,4,5,6,7]. While diatoms are rich in EPA, dinophyta are instead particularly rich in DHA. However, fatty acid composition and prevalence depends on the environmental conditions in which the microalgae live, such as temperature, light, macro- and micronutrients availability [8]. The importance of these PUFAs is also related to their function as precursors of eicosanoids, lipid mediators involved in many physiological processes such as defence, signal transduction, allelopathic competition, predator-prey interactions [9,10]. Prostaglandins are synthesized by the action of prostaglandin G/H synthase (PGHS), also known as cyclooxygenase (COX), that transforms fatty acid precursors, through two enzymatic steps, firstly into the highly unstable hydroperoxide prostaglandin G_2_ (PgG_2_), which is then rapidly reduced to prostaglandin H_2_ (PgH_2_). PgH_2_ is transformed in turn by specific synthases, namely prostaglandin E, F, D and I synthases (mPTGES, PTGFS, PTGDS and PTGIS) into prostaglandin E_2_, F_2α_, D_2_, I_2_ (PgE_2_, PgF_2α_, PgD_2_, PgI_2_), respectively [11]. PgE_2_ is transformed by the action of isomerases into PgA_2_ that is then converted to prostaglandin C_2_ and can be successively converted to PgB_2_. In addition, PgE_2_ can be reduced to PgF_2α_ by the 9-oxo-reductase [12]. PgJ_2_, instead, derives from dehydration of PgD_2_ [13]. Depending on which precursor is used, ETrA, ARA or EPA, the Pgs produced belong to series 1, 2 or 3, respectively [14].

While their involvement in the physiology of terrestrial organisms have been extensively studied, in marine organisms only scattered studies exist that have established their presence but not yet their function [15]. Very recently, the synthesis of Pgs have been investigated in phytoplanktonic organisms, such as marine diatoms [16,17]. The same Pgs molecules present in terrestrial animals were identified in the centric diatom species *Skeletonema marinoi* and *Thalassiosira rotula.* Interestingly, the biosynthetic pathway was found to be differentially expressed during the different growth phases of the cultures, and in the case of *Thalassiosira rotula,* the COX gene was differentially expressed between cultures growing in different concentrations of silica [17]. Studies on the evolutionary relationships of the diatom COX protein sequence with those from other organisms, showed similarity between diatom and animal COXs. Alignment of the diatom and human sequences denoted a 29% identity and a dendrogram construction showed the clustering of the diatom and human COX sequences in sister clades [15]. The above results indicate the conservation of the biosynthetic pathway for these mediators among the different kingdoms of life, suggesting an important ecological and functional role also in simple organisms. 

In mammalians, Pgs regulate important functions such as inflammation, tissue repair and immune response, and have, therefore, attracted much attention in view of high potentials as therapeutic agents. But the supply of Pgs from natural sources is difficult, and at the moment only chemical synthesis allows for their large-scale production. However, chemical synthesis is very expensive and new cheaper methods are needed for the pharmaceutical market [18].

Dinoflagellates, similarly to diatoms, are known to synthesize secondary metabolites that regulate their interactions with other microorganisms [7], which renders them very appealing as natural sources of bioactive molecules [19]. However, because of their toxicity and the tricky growing conditions they need, obtaining high biomass is difficult. Only one non-toxic species is currently grown at the industrial scale for commercial purposes, *Crypthecodinium cohnii*, a species producing high quantities of DHA [20]. Besides DHA, dinoflagellates are known to also produce high levels of eicosapentaenoic acid (EPA) [21], while arachidonic acid (ARA) is mostly absent.

Despite the high amount of EPAs produced, one of the precursors in the biosynthesis of Pgs, to the best of our knowledge, there are no investigations on the presence of the Pg pathway in this important group of microalgae. 

The high PUFA content and the growing interest about the setting-up of dinoflagellate growth conditions to produce bioactive molecules for industrial purposes [19,22], prompted us to perform a deep transcriptome mining of all the dinoflagellate sequenced transcriptomes available in the Marine Microbial Eukaryotes Transcriptome Sequencing Project (MMETSP) iMicrobe database [23]. Here we present the results of mining of these transcriptomes highlighting the presence of enzymes involved in Pgs synthesis and metabolism in this interesting microalgal group. We also present a comparative analysis to investigate the presence and relative expression of enzymes related to Pg metabolism in transcriptomes obtained under different growth conditions for the same clone or species.

## 2. Results

### 2.1. Dinoflagellate Transcriptomes 

The forty-two dinophyta transcriptomes available in the iMicrobe database have all been considered for data mining, covering 15 genera, 19 species and different combinations of growth conditions listed in Table 1.

The 42 transcriptomes differed in terms of number of assembled sequences not only among species but also among replicates of the same species or clone.

The assemblies’ sizes spanned from only 380 sequences for the species *A. sanguinea* to 106,664 sequences for *A. tamarense*-MMETSP0384 (Table 2). 

The species A. tamarense, K. brevis, strain Wilson and CCMP2229, and L. polyedra showed, even among replicates, different size assemblies. A. tamarense replicates spanned from 8411 to 61,753 transcripts, K. brevis strain Wilson MMETSP0201 and 0202 differed by 11,600 transcripts, the four replicates of K. brevis strain CCMP2229 spanned from 2594 to 11,371 transcripts, and L. polyedra replicates from 709 to 2423 transcripts.

These differences, even if not very large in terms of percentages of the total number of transcripts per transcriptome, are still relevant as thousands of sequences can represent intra-clone hidden functions and underline the necessity to perform the sequencing of a larger number of replicates. 

### 2.2. Prostaglandin Enzyme Identification

The search for the term “prostaglandin” in the Swiss-Prot annotation tables, identified nine Pgs metabolism-related functions. The identified functions were: Prostaglandin G/H synthase 2 (PTG/HS2 or COX2), prostaglandin E synthase 2 (PTGES2), hematopoietic prostaglandin D synthase (HPGDS), prostaglandin-E(2) 9-reductase (PGE_2_-9-OR), prostaglandin F synthase 1 and 2 (PTGFS1 and PTGFS2), 15-hydroxyprostaglandin dehydrogenase [NAD^+^] (15-PGDH), and prostaglandin reductase 1 and 2 (PTGR1 and PTGR2). The functions annotated as COX2, PTGES2, HPGDS, PGE_2_-9-OR, PTGFS1 and PTGFS2 are associated to enzymes involved in Pgs synthesis, while the ones annotated as 15-PGDH, PTGR1 and PTGR2 are associated to enzymes involved in Pgs catabolism [24,25]. The presence of both synthesising and catabolising Pgs-related functions strongly support the existence of an active Pgs metabolism in dinoflagellates. Overall, fourteen out of 19 species and 26 out of 42 transcriptomes had at least one annotated function related to Pg metabolism (Table 3). 

None of the selected transcriptomes expressed the complete set of identified functions (Table 4). The assemblies related to *K. brevis* and *G. foliaceum* were the ones expressing the majority of functions, since six of the nine enzymes involved in Pgs synthesis were annotated (Table 4). 

PTGES2, HPGDS, PGE_2_-9-OR, PTGFS1 and PTGFS2 were widely annotated among the transcriptomes, while COX2, the enzyme that executes the initial step for the synthesis of Pgs, was annotated only in two species, *A. massartii* and *P. glacialis* (Table 4). This result suggests that COX2 may need special conditions to be expressed at detectable levels. 

### 2.3. Clustering of Transcripts Associated to the Pgs-Related Enzymes 

Most of the annotated Pgs-related enzymes were associated to more than one transcript.

To exclude redundant sequences inside the transcriptomes, all the transcripts associated to the Pg pathway were analysed with the CD-Hit software. 

The analysis of all the transcripts of all the species altogether retrieved 102 clusters. Each cluster was very specific including only transcripts having the same function and coming from the same species, confirming the species specificity of each gene function and sequence equality of each transcript among different strains of a species.

The analysis was also performed for each species separately leading to the exclusion of the redundant transcripts (Table 5) and to the identification, in some species, of more than one independent protein, and thus genes, for HPGDS, PTGES2, PTGR1 and PTGR2 (Table 5). 

**Figure 1:**
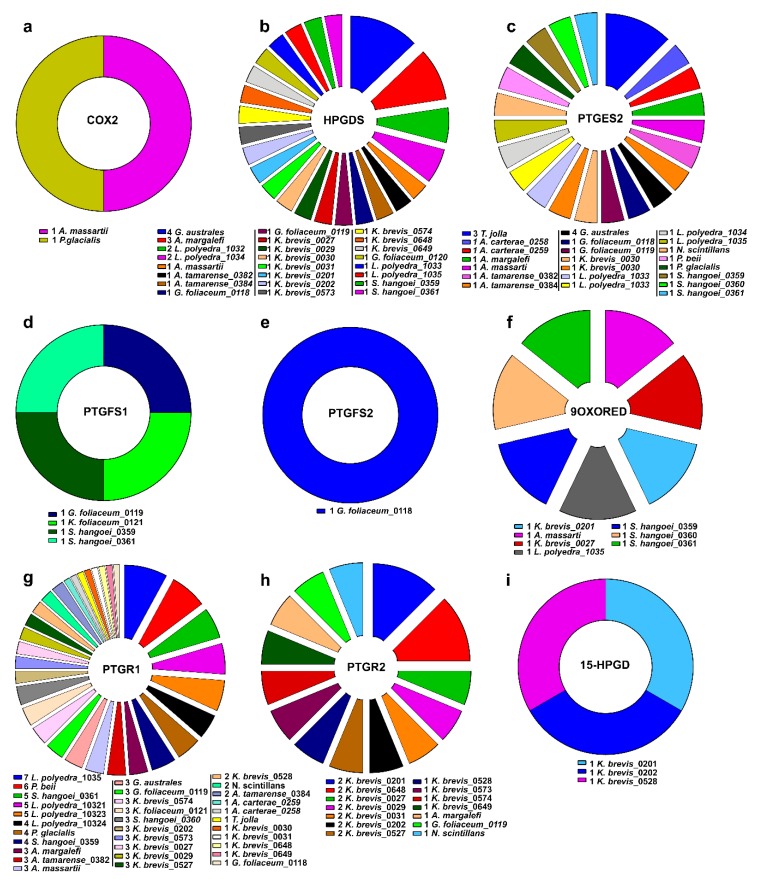
Occurrence of Pgs-related functions in each dinoflagellate species.

According to these analyses, HPGDS, PTGES2 and PTGR1 were the most widespread functions in almost all the species considered (Figure 1).

### 2.4. In-Silico Analysis of Expression Levels

**Figure 2:**
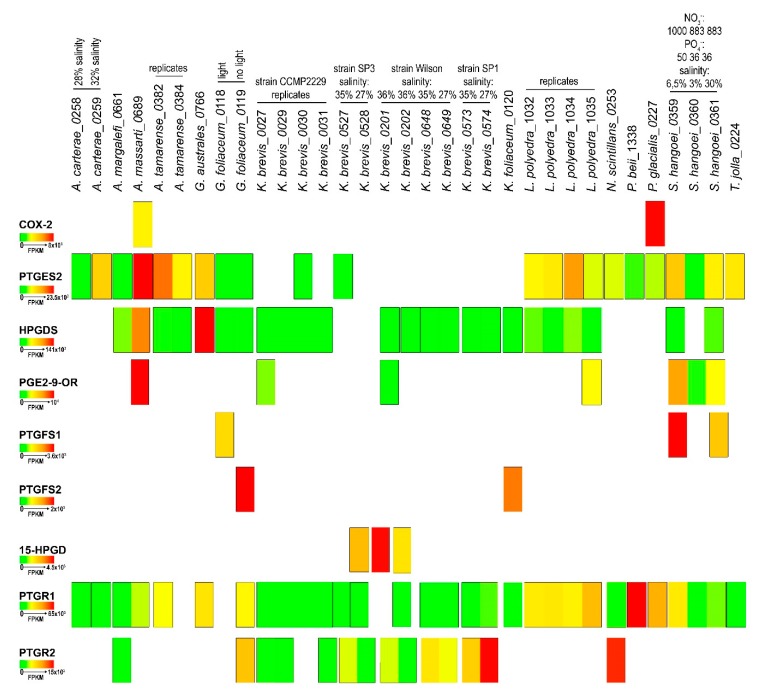
Transcript expression of the prostaglandin-related functions. Heat map representation of the expression levels, in Fragments per Kilobase per Million Mapped Reads (FPKM), of the Pgs metabolism related enzymes identified in each transcriptome of the different dinoflagellate species.

The in-silico expression analysis of the selected transcript for each species showed a large variability in expression levels both among different strains of the same species, culture replicates and different growing conditions. Figure 2 highlights the differences among species in relation to Pg functions, based on Fragments Per Kilobase per Million mapped reads (FPKM) values calculated for each transcriptome.

The results indicated that the COX2 transcript showed the greatest expression in *P. glacialis*. On the other hand, PTGES2, widely expressed in the species analysed, showed the highest FPKM value in *A. massartii*. Interestingly, in *K. brevis*, of the four different strains and two replicates for each condition, PTGES2 was expressed only in one replicate for each of the strains CCMP2229 and SP3. HPGDS expression was very low and at similar expression levels in all species except for *G. australes* and *A. massartii* (Figure 2). PGE_2_-9-OR was highly expressed in *A. massartii*, which also showed the highest expression with respect to other species such as CCMP2229 and Wilson *K. brevis* strain, *L. polyedra* and *S. hangoei*. PTGFS1 and PTGFS2 were found expressed only in three species, *G. foliaceum*, *K. foliaceum* and *S. hangoei* with highest values of PTGFS1 in *S. hangoei* and highest values of PTGFS2 in *G. foliaceum*. 15-HPGD was expressed only in the Wilson and SP3 strains of *K. brevis*, with one replicate of the Wilson strain having a higher expression with respect to one replicate of the strain SP3. PTGR2 was expressed only in *A. margalefi*, *G. foliaceum*, *N. scintillans* and some strains of *K. brevis*, one of which showed the highest expression value. PTGR1 instead was more expressed in *P. beii* with respect to all other species that showed comparable expression levels. 

**Figure 3:**
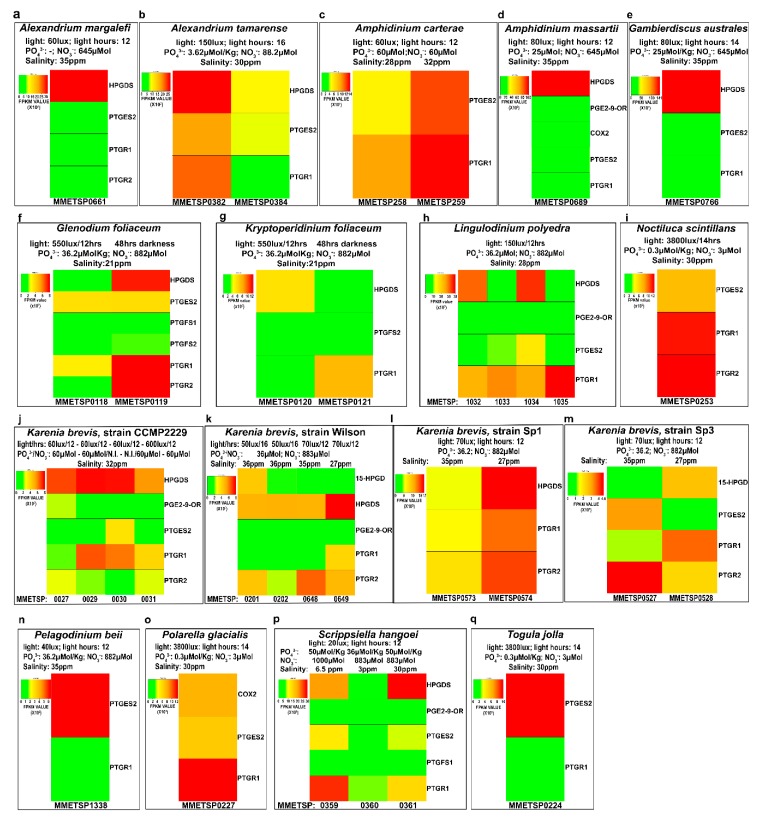
Pg-related transcript expression for each species. The heat maps represent the expression of each Pg-related transcript, expressed in Fragment Per Kilobase per Million mapped reads (FPKM), for each species, grouping the strains and culture conditions.

Figure 3 reports the gene expression levels of each Pg function in each transcriptomes grouped according to the species and replicates. These analyses highlighted a lack of reproducibility in the expression levels among replicates (*A. tamarense*, *L. polyedra*, *K. brevis*-strain CCMP2229, *K. brevis*- Wilson strain, Figure 3).

Overall, HPGDS, when present, was the most expressed function (Figure 3a,b,d,e,f,h,j,l,p) with respect to the others. 

Different light conditions induced differential expression of the Pgs-related transcripts in *G. foliaceum* and its basionym *K. foliaceum,* in which the different light conditions of growth showed appreciable differences in terms of both genes expressed and intensity of expression (Figure 3f,g). 

*K. brevis* is one of the best represented species in the database, being present with 12 transcriptomes for four strains. Unfortunately, we could not extrapolate very good information from these 12 transcriptomes since, even among equal replicates of the same strain (Figure 3j, strain CCMP2229), reproducibility was not appreciable both as type of gene expressed and level of expression. In *K. brevis* strain SP1, lower salinity percentages seemed to induce higher expression of HPGDS, PTGR1 and PTGR2 (Figure 3l), while in *K. brevis* strain SP3 PTGES2 and PTGR2 are downregulated in conditions of lower salinity (Figure 3m).

*S. hangoei* (Figure 3p) showed differences among different salinity treatments, while the genus *Amphidinium* (Figure 3c,d) showed differences between the two species analysed, *A. carterae* and *A. massartii*. *A. carterae* (Figure 3c), in both conditions expressed only two genes, PTGES2 and PTGR1, while *A. massartii* (Figure 3d) expressed almost the complete set of genes including COX2 and the enzymes for PgE_2_, PgF_2α_, PgD_2_ synthesis and degradation (PTGR1).

The genus *Alexandrium* (Figure 3a,b) showed variability among equal replicates and differences among different species of the same genus (i.e., *A. margalefi* (Figure 3a) and *A. tamarense* (Figure 3b)).

All four *L. polyedra* replicates had expression of HPGDS, PGE_2_-9-OR, PTGES2 and PTGR1, except for PGE_2_-9-OR. In addition, in this case, the data showed no reproducibility (Figure 3h). 

*S. hangoei* at higher salinity conditions had higher HPGDS expression while higher PTGR1 expression at lower percentage salinity (Figure 3p).

## 3. Discussion

Prostaglandins are key physiological mediators involved in many important physiological processes in animals. In terrestrial animals, they have been widely studied and their role in inflammation, development, pregnancy, sexual reproduction and defence have been established and correlated to the three series of Pgs molecules [26]. 

Different types of Pgs have been discovered also in marine organisms but their role in the marine environment still awaits to be discovered [15].

Recently, we identified a wide set of Pgs in diatoms—eukaryotic unicellular microalgae widely distributed in most aquatic environments. This discovery was surprising, as their presence in the vegetal kingdom is still not ascertained and their discovery in so “simple” and ancient organisms posed interesting ecological and evolutionary questions [16].

Apart from diatoms, among the phytoplanktonic microalgae, dinoflagellates are important primary producers in the marine food webs. As other microalgae, they are good PUFA producers, particularly of the ω3 type DHA [7,8,20]. 

Dinoflagellates are well recognised as producers of very powerful bioactive molecules. Some examples include macrolides, cyclic polyethers, spirolides and purine alkaloids, fatty acids, pigments and polysaccharides that strongly affect biological receptors and metabolic processes, such as inflammation, pain, infection and others [19]. In addition, dinoflagellates are one of the most important microalgal groups responsible for the occurrence of harmful algal blooms (HABs). During HABs they produce potent toxins such as saxitoxins, gonyautoxins, brevetoxins, yessotoxins, ciguatoxins, maitotoxins, azaspiracid toxins, and palytoxin [27]. Although these toxins can cause severe poisoning, they may have interesting pharmacological applications due to their chemical structure and mechanisms of action [27]. 

There is a current urgent need for antibiotics, anti-cancer and anti-inflammatory agents and dinoflagellates may provide new compounds to meet these needs [19,28]. The genus *Amphidinium* for example is a good producer of macrolides and polyketides with cytotoxic activity against tumour cell lines [29,30]. *Karlodinium veneficum* is another good example of species useful for pharmaceutical purposes, since it produces a group of toxic metabolites named karlotoxins with haemolytic, cytotoxic and ichthyotoxic activity [28]. Their mechanism of action is based on the formation of pores in the cell membrane that destroys the osmotic balance of the cells leading to cell death [28]. This cell membrane pore formation is being used to develop a new pharmacophore anti-cholesterol therapy [28].

In this context of urgent need for new biologically active molecules, the use of omics technologies applied to the discovery of new natural products from simple marine microorganisms is growing day by day. Numerous studies demonstrate the reliability of RNA-seq to find secondary metabolic pathways for potent bioactive molecules both in terms of gene sequence accuracy and level of their expression [23,31]. From this point of view, dinoflagellates are still a “hard” subject to work on because of their large and very structurally complex genomes (elevated amount of introns, repetitive redundant non-coding sequences, unusual bases, lack of recognizable promoter features and typical eukaryotic transcription factors) that render the identification of bioactive-molecules-related genes very complex [19]. Indeed, only one genome has been partially sequenced until now, the one of *Symbiodinium minutum.* Nonetheless, omics technologies, particularly proteomics and transcriptome sequencing, are now being applied to identify toxin related genes [32,33,34,35]. The Moore foundation has helped this process by funding the sequencing of many dinoflagellate transcriptome species [23].

**Figure 4:**
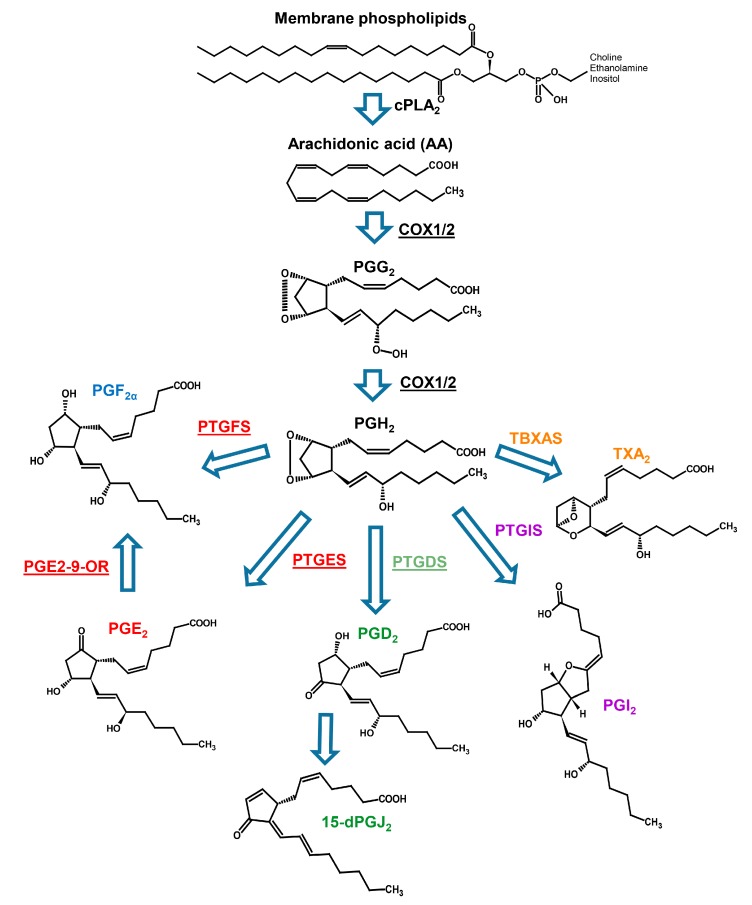
Prostaglandin biosynthetic pathway. Enzymes involved in the pathway are reported as underlined text next to the arrows. For the abbreviation, refer to the text (Modified from Di Costanzo et al. 2019 [15]).

Using the sequencing approach coupled to deep analysis level of data mining, we have been able to find also in dinoflagellates the enzymes involved in prostaglandin biosynthesis (Figure 4). 

Our results suggest that dinoflagellates have the potential to synthesize at least three types of Pgs, namely PgE, PgD and PgF_2α_, due to the presence of prostaglandin E_2_, D_2_, F_1_ and F_2_ synthases, although COX is expressed at detectable levels only in two species, *A. massartii* and *P. glacialis*. The presence in many species of enzymes reducing PgE_2_, PTGR1 and PTGR2 and 15-HPGD, suggests that PgE_2_ may be synthesized even in the absence of proper cyclooxygenase activity. Indeed, in the diatom *Phaeodactylum tricornutum*, Pgs are synthetized in an enzyme independent manner [36], suggesting that this could also be the case for dinoflagellate species in which the expression of COX transcripts is not detectable. On the other hand, a very low level of expression, undetectable by sequencing methods adopted, can be the reason for the absence of the COX transcripts. This was the case for a *S. marinoi* strain, named FE60, that, with respect to another strain, named FE7, had an RNA-seq undetectable level of COX expression that was conversely detectable by qPCR technique [16].

In-silico expression analysis reported in the present work revealed interesting differences among different strains of the same species grown in similar (e.g., *K. brevis* strains) or different conditions (i.e., light versus dark; nutrient depletion; salinity). These results further demonstrate the capability of different strains of a same species, to differently express secondary metabolites [7].

Despite sequencing technology is now robust and reliable, some remarks are warranted on the potential results that are obtained from the in-silico analysis of transcriptomes. From our analysis, assemblies’ size of the transcriptomes analysed was different even among replicates of the same strain. This could be due to the occurrence of intra-clone hidden functions and could be the reason why we find the COX2 function annotated only in two species despite the annotation of the other synthetic and reducing functions of Pgs. These observations underline the necessity to sequence a statistically sufficient number of replicates to obtain robust results from bioinformatic analysis. Finally, experimental approaches to study gene expression and chemical identification of Pgs are needed to confirm the functionality of Pg pathway in dinoflagellates.

## 4. Materials and Methods 

### 4.1. Transcriptomes Collection

The iMicrobe (https://www.imicrobe.us/#/projects/104) database has been questioned with the word “Dinoflagellate” to select all the sequenced transcriptomes in this group of microalgae.

Nt.fa. pep.fa, swissprot.gff3, cds.dat, contig.dat and stats.txt file were downloaded for each species listed in Table 1. Attributes listed in each assembly page were taken to create the physical-chemical information provided in Table 1. The stats.txt files were applied to extrapolated transcriptome sequences statistics to create Table 2.

### 4.2. Prostaglandin Enzymes Identification

The term “prostaglandin” was used to query the swissprot tables of each transcriptome.

The corresponding transcript ID of each species and of each Pgs-related function were listed and counted to create Table 3 and Table 4 and Figure 1.

### 4.3. Gene Clustering

All the id corresponding to prostaglandin functions were used to retrieve the corresponding peptide sequences from the pep.fa files. CD-Hit software (http://weizhongli-lab.org/cd-hit/) was used to cluster and compare the repetitive sequences of each transcript associated to a Pg-related function. Sequences considered head of the cluster were used for the following analysis.

### 4.4. Gene Expression Analysis

Gene expression levels were calculated using the contig.dat reads data using the fragments per kilobase per million mapped reads (FPKM) formula: [mapped reads pairs]/([length of transcript]/1000)/([total reads pairs]/10^6^). If more than one transcript per Pg-related function was present, the corresponding FPKM values were added together, considering that the level of expression of a gene derives from the sum of all the genes expressing the same function at the same moment. Expression levels were represented by heat map function calculated using R scripts (https://www.r-project.org/).

## 5. Conclusions

The transcriptome mining approach used in this work succeeded in revealing the presence of Pg pathway in 14 out of 19 dinoflagellate species analysed. Only *K. brevis* possesses an almost complete set of enzymes involved in the pathway, although no annotation for COX was identified. This enzyme, catalysing the initial step of Pg biosynthesis, was found annotated only in two species, *A. massartii* and *P. glacialis*. The CD-HIT analysis excluded transcript redundancy (Table 5) and identified, in some species, more than one independent protein for HPGDS, PTGES2, PTGR1 and PTGR2 (Table 5). 

In-silico analysis of gene expression levels showed differences only in *G. foliaceum* under different light conditions, while in *S. hangoei* HPGDS and PTGR1 were differentially expressed in different salinity conditions.

These findings pave the way for future investigations through experimental gene expression analysis and chemical identification, in order to confirm Pgs presence in dinoflagellates and stimulate further researches toward the understanding of their ecological role in nature.

## Figures and Tables

**Table 1:** List of dinoflagellate species present in the iMicrobe database. For each species, physical-chemical growth conditions and the geographical area from which they have been isolated are reported. Table abbreviations: Day portion in hours: Light portion of the day/night cycle in hours; Exp: Experimental; N.I.: Not indicated; GEO: Geographical; MMETSP: Marine Microbial Eukaryotes Transcriptome Sequencing Project.

Genus	Species	MMETSP Code	Light	Day Portion in hours	Phosphate µmol/kg	Nitrate µMol	Exp. Salinity (%)	Note	Geo. Area
*Akashiwo*	*sanguinea*	223	3800	14	0.3	3	30		New York
*Alexandrium*	*fundyense*	0196C	200	14	36.2	882	28		Atlantic Ocean
	*margalefi*	661	60	12		645	35		Tasmania
	*minutum*	328	100	12	36.2	882	25		Ria da Vigo
	*tamarense*	380	150	16	3.62	88.2	30		English Channel
		378	150	16	3.62	88.2	30		English Channel
		384	150	16	3.62	88.2	30		English Channel
		382	150	16	3.62	88.2	30		English Channel
*Amoebophrya*	*-*	795	200	14	36.2	882	25		-
*Amphidinium*	*carterae*	258	60	12	60	60	28		Massachusetts USA
		259	60	12	60	N.I.	32		Massachusetts USA
		0398C	200	14	36.2	882	32		Atlantic Ocean
	*massartii*	689	80	12	25	645	35		Pacific Ocean
*Gambierdiscus*	*australes*	766	80	14	25	645	35		Pacific Ocean
*Glenodinium*	*foliaceum*	118	550	12	36.2	882	21	Antibiotic	Chesapeake Bay
		119		48hr dark	36.2	882	21		Chesapeake Bay
*Karenia*	*brevis*	201	50	16	36	883	36	Strain Wilson	Gulf of Mexico
		202	50	16	36	883	36		Gulf of Mexico
		648	70	12	36.2	882	35		Gulf of Mexico
		649	70	12	36.2	882	27		Gulf of Mexico
		27	60	12	60	60	32	Strain CCMP2229	
		29	60	12	60	N.I.	32		
		30	60	12	N.I.	60	32		
		31	600	12	60	60	32		
		573	70	12	36.2	882	35	Strain SP1	
		574	70	12	36.2	882	27		
		527	70	12	36.2	882	35	Strain SP3	
		528	70	12	36.2	882	27		
*Kryptoperidinium*	*foliaceum*	120	550	12	36.2	882	21	Antibiotic	California USA
		121		48 h dark	36.2	882	21	Antibiotic	California USA
*Lingulodinium*	*polyedra*	1034	150	12	36.2	882	28		Gulf of Mexico
		1033	150	12	36.2	882	28		Gulf of Mexico
		1032	150	12	36.2	882	28		Gulf of Mexico
		1035	150	12	36.2	882	28		Gulf of Mexico
*Noctiluca*	*scintillans*	253	3800	14	0.3	3	30		Puget Sound, WA
*Pelagodinium*	*beii*	1338	40	12	36.2	882	35		Caribbean Sea
*Polarella*	*glacialis*	227	3800	14	0.3	3	30		McMurdo Sound
*Pyrocystis*	*lunula*	229	3800	14	0.3	3	30		-
*Scrippsiella*	*hangoei*	359	20	12	50	1000	6.5	Selenium 4.55 nMol/L	Baltic Sea
		360	20	12	36	883	3		Baltic Sea
		361	20	12	36	883	30		Baltic Sea
*Togula*	*jolla*	224	3800	14	0.3	3	30		

**Table 2:** Sequencing statistic data of the dinoflagellate transcriptomes deposited in the iMicrobe database.

Species	Genus	MMETSP Code	Number of Sequences	Minimum Contig Length	Maximum Contig Length	N50
*Akashiwo*	*sanguinea*	223	380	150	593	197
*Alexandrium*	*fundyense*	0196C	7872	150	2586	273
	*margalefi*	661	54,023	150	7976	1055
	*minutum*	328	13,126	150	2630	823
	*tamarense*	380				
		378	44,911	150	5858	701
		384	106,664	150	13,283	1398
		382	98,253	150	9488	1506
*Amoebophrya*	*-*	795	16,699	150	35,302	3178
*Amphidinium*	*carterae*	258	44,378	150	13,731	1899
		259	45,656	150	9671	1781
		0398C	7775	150	1411	225
	*massartii*	689	53,416	150	11,086	1897
*Gambierdiscus*	*australes*	766	53,551	150	5938	1201
*Glenodinium*	*foliaceum*	118	80,537	150	8317	1259
		119	89,413	150	51,794	950
*Karenia*	*brevis*	201	89,316	150	24,712	1642
		202	77,716	150	23,631	1298
		648	83,137	150	28,240	1563
		649	87,365	150	24,093	1566
		27	87,338	150	20,088	1514
		29	88,007	150	17,512	1524
		30	90,601	150	15,457	1493
		31	79,230	150	11,278	1499
		573	91,547	150	24,219	1728
		574	99,942	150	19,522	1792
		527	81,513	150	14,879	1664
		528	82,936	150	18,526	1658
*Kryptoperidinium*	*foliaceum*	120	93,725	150	13,985	1627
		121	80,158	150	5242	1190
*Lingulodinium*	*polyedra*	1034	87,027	150	9207	1260
		1033	87,335	150	15,159	1339
		1032	89,450	150	11,782	1331
		1035	88,741	150	22,209	1427
*Noctiluca*	*scintillans*	253	45,249	150	11,484	1594
*Pelagodinium*	*beii*	1338	55,559	150	15,296	1545
*Polarella*	*glacialis*	227	74,437	150	23,079	1587
*Pyrocystis*	*lunula*	229	572	150	1058	198
*Scrippsiella*	*hangoei*	359	81,854	150	16,784	1701
		361	83,907	150	33,299	1595
		360	69,908	150	12,551	1281
*Togula*	*jolla*	224	47,727	150	8640	1570

**Table 3:** Number of species and transcriptomes in which each prostaglandin metabolism related function was found annotated.

Enzyme	N° of Species	N° of Transcriptomes
Cyclooxygenase (COX2)	2	2
Hematopoietic prostaglandin D synthase (HPGDS)	9	18
Prostaglandin E synthase 2 (PTGES2)	12	20
15-hydroxyprostaglandin dehydrogenase [NAD^+^] (15-PGDH)	1	2
Prostaglandin-E(2) 9-reductase (PGE_2_-9-OR)	4	6
Prostaglandin F synthase 1 (PTGFS1)	3	4
Prostaglandin F synthase 2 (PTGFS2)	1	1
Prostaglandin reductase 1 (PTGR1)	14	24
Prostaglandin reductase 2 (PTGR2)	4	7

**Table 4:** List of the fourteen species presenting annotated enzymes related to prostaglandin metabolism. The occurrence of each enzyme per species is indicated. Abbreviations: Hematopoietic prostaglandin D synthase: HPGDS; prostaglandin reductase 1: PTGR1; prostaglandin E synthase 2: PTGES2; prostaglandin-E(2) 9-reductase: PGE_2_-9-OR; prostaglandin G/H synthase 2: PTG/HS2 (COX2); prostaglandin F synthase 1: PTGFS1; prostaglandin F synthase 2: PTGFS2; prostaglandin reductase 2: PTGR2; 15-hydroxyprostaglandin dehydrogenase [NAD+]: 15-PGDH.

	PTG/HS2 (COX2)	HPGDS	PTGES2	PGE2-9-OR	15-PGDH	PTGFS1	PTGFS2	PTGR1	PTGR2
*A. margalefi*		X						X	X
*A. tamarense*		X	X					X	
*A. carterae*			X					X	
*A. massartii*	X	X	X	X				X	
*G. australes*		X	X					X	
*G. foliaceum*		X	X			X	X	X	X
*K. brevis*		X	X	X	X			X	X
*K. foliaceum*		X				X		X	
*L polyedra*		X	X	X				X	
*N. scintillans*			X					X	X
*P. beii*			X					X	
*P. glacialis*	X		X					X	
*S. hangoei*		X	X	X		X		X	
*T. jolla*			X					X	

**Table 5:** CD-Hit clustering of the transcripts associated to each Pgs-related function. Table abbreviations: SP: Number of transcripts associated to each function by blast against the Swiss Prot database; CD-Hit: Number of genes associated to each function after clustering analysis. Number in merged cells indicated that before and after the clustering the gene number did not change.

Species	MMETSP	15-HPGD		9OXORED		HPGDS		PTGES2		PTGFS1		PTGFS2		PTG/HS2		PTGR1		PTGR2	
		SP	CD	SP	CD	SP	CD	SP	CD	SP	CD	SP	CD	SP	CD	SP	CD	SP	CD
			Hit		Hit		Hit		Hit		Hit		Hit		Hit		Hit		Hit
*A. margalefi*	661	-		-		3		1		-		-		-		4	3	1	
*A. tamarense*	382	-		-		1		1		-		-		-		4	3	-	
	384	-		-		1		1		-		-		-		2		-	
*A. carterae*	258	-		-		-		2	1	-		-		-		2	1	-	
	259	-		-		-		1		-		-		-		1		-	
*A. massartii*	689	-		1		3	1	1		-		-		2	1	3		-	
*G. australes*	766	-		-		4		1		-		-		-		3		-	
*G. foliaceum*	118	-		-		1		1		-		1		-		1		-	
	119	-		-		1		2	1	1		-		-		3		1	
*K. brevis*	201	1		1		1		-		-		-		-		-		3	2
	202	1		-		1		-		-		-		-		2		1	
	648	-		-		2	1	-		-		-		-		1		2	
	649	-		-		1		-		-				-		1		1	
	573	-		-		1		-		-		-		-		2		1	
	574	-		-		1		-		-		-		-		4	3	1	
	27	-		1		1		-		-		-		-		2		1	
	29	-		-		1		-		-		-		-		3	2	2	1
	30	-		-		1		1		-		-		-		1		-	
	31	-		-		1		-		-		-		-		1		1	
	527	-		-		-		1		-		-		-		2		2	1
	528	1		-		-		-		-		-		-		2		1	
*K. foliaceum*	120	-		-		1		-		-		-				-		-	
	121	-		-		-		-		1		-		-		3		-	
*L. polyedra*	1032	-		-		4	2	3	1	-		-		-		8	5	-	
	1033	-		-		1		2	1	-		-		-		6	5	-	
	1034	-		-		3	2	2	1	-		-		-		5	4	-	
	1035	-		1		1		3	1	-		-		-		10	7	-	
*N. scintillans*	253	-		-		-		1		-		-		-		2		1	
*P. beii*	1338	-		-		-		1		-		-		-		6		-	
*P. glacialis*	227	-		-		-		1		-		-		4	1	6	4	-	
*S. hangoei*	359	-		1		1		1		1		-		-		4		-	
	360	-		1		-		1		-		-		-		3		-	
	361	-		1		1		1		1		-		-		5		-	
*T. jolla*	224	-		-		-		3		-		-		-		2	1	-	
